# Interplay between Body Size Measures and Thyroid Cancer Aggressiveness: A Retrospective Analysis

**DOI:** 10.1155/2018/2089471

**Published:** 2018-08-27

**Authors:** Thaís Gomes de Melo, Ligia Vera Montali da Assumpção, Denise Engelbrecht Zantut-Wittmann

**Affiliations:** Division of Endocrinology, Department of Internal Medicine, University of Campinas, Campinas, Brazil

## Abstract

Considering controversial data about the relationship between body size and prognosis of differentiated thyroid cancer (DTC), the current study aimed to assess the influence of body weight, body mass index (BMI), and body surface area (BSA) on DTC. We conducted a retrospective analysis of patients' records from the Thyroid Cancer Unit, assessing body size measures, clinical and laboratory prognostic factors, and disease evolution. 337 patients, aged 45.95 ± 13.04 years old, with BMI of 27.87 ± 5.13 kg/m^2^ and BSA of 1.74 ± 0.18 m^2^ were enrolled. After 9.5 ± 6.9 years of follow-up, 87.29% of patients were disease-free and 12.71% had persistent disease; no patient had deceased. Patients aged <45 years old with extrathyroidal invasion tumor had greater baseline body weight and BSA than those without extrathyroidal invasion (median 79.5 kg versus 67 kg and 1.85 m^2^ versus 1.74 m^2^). Women with poorly differentiated tumor and patients aged ≥45 years old with distant metastasis presented greater weight loss during follow-up compared to patients without such characteristics (median −2 kg versus +1.5 kg and −3 kg versus +1 kg, respectively). The relationship between body size and DTC evolution was not observed. In conclusion, higher weight and BSA were associated with a greater chance of extrathyroidal tumor invasion in younger patients. Specific subgroups of patients with aggressive disease presented higher weight loss. Young patients with higher BSA should be carefully treated due to possible worse prognosis related to increased incidence of extrathyroid invasion. Findings related to tumor aggressiveness and weight loss in specific groups deserve further mechanistic studies.

## 1. Introduction

Differentiated thyroid cancer (DTC) is a neoplasia with raising incidence and patients usually present long survival [1, 2]. Overweight and obesity are also increasing in prevalence [3] and are related to a higher incidence of at least 17 different types of cancer, including thyroid cancer [4]. Excess of body weight influences tumor genesis through the effects of different hormones and cytokines, such as insulin, interleukins, and tumor necrosis factor alpha [5, 6]. The same factors may be implicated in the occurrence of more aggressive malignant tumors [5, 6]. Higher body mass index (BMI) is associated with the development of tumors with characteristics of higher aggressiveness [5, 7] and also to higher cancer-related mortality in different kinds of malignant neoplasias [8]. However, such relationship of body size measures and worse prognosis and evolution of disease is controversial in thyroid cancer [9–11].

Some publications suggested an association of higher BMI and characteristics of thyroid tumor aggressiveness. Trésallet et al. [11] evaluated papillary thyroid cancer (PTC) patients followed by 6 years, and BMI was identified as an independent risk factor for local disease recurrence only for tumors sized ≥1 cm. In agreement with that, a study published by Kim et al. [12] reported that increment in BMI was associated with higher frequency of PTC nodules of ≥1 cm, extrathyroid invasion, and more advanced tumor stage. Nevertheless, after mean follow-up of 84 months, there were no differences in recurrence rates according to BMI range [12]. Other studies could not prove an association between BMI and characteristics of worse prognosis, such as a retrospective analysis by Paes et al., which showed no positive relationship between initial BMI, tumor vascular invasion or persistence, and recurrence of DTC in DTC patients followed for 6.2 years [10]. Other controversial results were shown by Kim et al., evidencing overweight and obesity as independent risk factors for tumor multiplicity and extrathyroid invasion in women, but not for the occurrence of the bilateral tumor and lymph nodes metastasis [9]. In addition, Grani et al. showed no significant correlation between BMI, primary tumor size, and American Thyroid Association initial risk stratification system score and no single feature of advanced thyroid cancer was more frequent in obese patients than in others. But interestingly, BMI was a predictor of microscopic extrathyroidal extension only [13].

Considering obesity is associated to higher incidence of malignant tumors and also to worse prognosis of different kinds of neoplasia, but there are no conclusive data regarding DTC, the current study was performed with the objective of evaluating the impact of body size measures in prognostic factors and evolution of disease, involving a greater number of different clinical and histological parameters and longer follow-up compared to previously published studies.

## 2. Materials and Methods

This was a retrospective analysis of the hospital files from the Thyroid Cancer Unit of the University of Campinas. All patients were submitted to total thyroidectomy and were treated according to thyroid cancer guidelines between 1980 and 2015 [14]. Patients who were followed for at least 12 months after initial surgical treatment and who had an appropriate report of height and body weight at diagnosis and during follow-up in their hospital files were included. For measuring weight, the hospital's 150 kg digital platform weigh scale (Filizola®), with an accuracy of weight to 0.1 kg, was used. Height was measured using a stadiometer linked to the scale (height rod). Trained hospital staff members were responsible for the measurements and registration in the records, and the weigh scale is professionally calibrated every year.

BMI (kg/m2) was calculated according to the guidelines from the World Health Organization using the formula weight (kg)/height (m) squared. Patients were categorized as normal weight (18.5 ≤ BMI < 25 kg/m^2^), overweight (25 ≤ BMI < 30 kg/m^2^), or obese (BMI ≥ 30 kg/m^2^) [15]. BSA was determined by the formula developed by DuBois and DuBois: BSA (m^2^) = 0.007184 × weight (kg)^0.425^ × height (cm)^0.725^ [16]. Since no consensus for BSA categorization exists, the patients were grouped into BSA quartiles by sex. Collected information about body size measures included initial and last register of weight, BMI, BSA, and weight change over time. Patients whose weight and height were not properly registered in their files at diagnosis and follow-up for any reason or who had not completed at least 12 months of follow-up were excluded from the analysis.

We also evaluated the following clinical and histological parameters of the cancer: age at diagnosis, ethnicity, family history of DTC, smoking history, follow-up time, presence of thyroid antibodies, size and number of malignant nodules, histology type and variant, vascular and lymphatic invasion, extracapsular or extrathyroidal invasion, histological differentiation grade, metastasis to lymph nodes and to distant organs, and tumor staging according to the tumor node and metastasis system (TNM) [17]. Presence of lymph nodes metastasis at diagnosis was considered positive according to the information after the first surgery, which was related to nodes dissection and nodes biopsy. Patients' status was classified at the last follow-up register in one of the following status: disease-free, persistent disease, recurrent disease, or death due to DTC [18]. Disease-free state was defined as TG < 1 ng/mL (radioimmunoassay (RAI) method), absence of antithyroglobulin antibodies (RAI method, positivity cutoff 115 mUI/L), and absence of abnormal findings in neck ultrasound, according to the guidelines of thyroid cancer management. Persistent disease was considered for the sustained occurrence of TG ≥ 1 ng/mL or image findings suggestive of the structural disease since the start of clinical follow-up. Recurrent disease was considered for patients who presented such features after being considered as disease-free in any previous point in time.

Data were tabulated, and we performed an exploratory analysis through summary measures (mean values, standard deviation, median, frequency, and percentage) in order to evaluate the impact of body size measures on parameters of DTC. Comparative analyses were performed by comparing median values of applicable variables using Mann–Whitney, Kruskal-Wallis, Chi-square, or Fisher exact tests. A significance level of 5% was considered significant in the analysis. All analyses involving anthropometry data were planned to be done by sex, because men and women's body composition is known to be different [19]. The split analysis between patients aged below and equal or superior to 45 year old was also planned, since it is the cutoff point of age to consider a worse prognosis in DTC [17]. The present study has been approved by the appropriate institutional ethics committee (Ethical Committee of the University of Campinas, under the certificate number 61613416.4.0000.5404). All procedures in the study were in accordance with the ethical standards of the institutional research committee and with the 1964 Helsinki declaration and its later amendments or comparable ethical standards. For this type of study, a formal consent is not required (waiver was approved by the Ethics Committee).

## 3. Results and Discussion

### 3.1. Results

From 939 patients in the Thyroid Cancer Unit's database, 337 presented all applicable anthropometry information in their files and were considered to the study. Majority (87.8%) of patients was female and had tumor histology of PTC (89.29%); mean age at diagnosis was 45.95 ± 13.04 years old. Mean initial BMI was 27.87 ± 5.13 kg/m^2^ and mean initial BSA was 1.37 ± 0.21 m^2^. One-hundred women (33.9%) had initial BMI < 25 kg/m^2^, 107 (36.1%) presented 25 ≤ BMI <30 kg/m^2^, and 89 (30%) had BMI ≥ 30 kg/m^2^. The respective numbers for male patients were 9 (21.9%), 20 (48.8%), and 12 (29.3%). After mean follow-up time of 9.5 ± 6.9 years, mean BMI increased to 28.51 ± 5.34 kg/m^2^ and mean BSA to 1.76 ± 0.18 m^2^. Regarding the evolution of the disease, it was not possible to get accurate information in 38 files. From the remaining 299, 87.29% of patients were classified as disease-free state and 12.71% as persistent disease state. No patients included in the analysis have died due to DTC or were classified as recurrent disease state. Detailed clinical and laboratory characteristics of patients are described in Tables 1 and 2.

The analysis according to age group evidenced patients aged less than 45 years old who presented tumors with extrathyroidal invasion had higher weight and BSA at diagnosis than those patients without such invasion (median of 79.5 kg versus 67 kg, *p* = 0.0253 and 1.85 m^2^ versus 1.74 m^2^, *p* = 0.0096, respectively). During follow-up, such difference was sustained, considering last assessment of weight and BSA (median of 80.9 kg versus 72 kg, *p* = 0.0307 and 1.89 m^2^ versus 1.76 m^2^, *p* = 0.0105, respectively). Such results are in line with the association of higher body measures and occurrence of more aggressive cancer. Female patients with poorly differentiated tumors have lost more weight compared to patients with well-differentiated tumors (median of −2 kg versus +1.5 kg of change over time, *p* = 0.0306) from the same gender. Also, patients aged 45 years old or more and who presented distant metastasis during follow up presented greater weight loss during evolution than patients of the same age without such metastasis (median of −3 kg versus +1 kg of weight change, *p* = 0.0370). This was an unexpected finding, which was not related to the initial aim of assessing the relationship between higher body size measures and tumor evolution. Those were the only significant findings, and they are shown in details in Table 3. All other analyses were nonsignificant.

The analysis of prognosis considering comparison of median values between disease-free and persistent disease groups of patients evidenced that the following variables were associated with higher occurrence of persistent disease: size of nodules (*p* = 0.021), follicular thyroid cancer (FTC) histology (*p* = 0.0030, versus PTC), distant metastasis at diagnosis or during evolution (*p* < 0.0001 for both), metastasis for lymph nodes at diagnosis (*p* = 0.0002), vascular and lymphatic tumor invasion (*p* = 0.0008), extracapsular invasion (*p* < 0.0001), and extrathyroidal invasion (*p* = 0.0036). The multivariate analysis of the same tumor factors showed only initial lymph nodes metastasis to be correlated with CDT prognosis (odds ratio = 11.4, CI 95% - 2.957–44.600, *p* = 0.004).

Height, initial and final weight, BMI, and BSA presented no relationship with prognosis. Considering BMI categories (normal weight, overweight, and obesity) by sex, there were no significant differences between disease-free patients group and persistent disease patients group (Table 4). Also, considering BSA quartiles by sex, we did not find any relationship with prognosis (Table 5). It is important to highlight that this analysis was possible to be done only with the 299 patients with complete follow-up information. Therefore, weight, BMI, and BSA did not impact on prognosis according to our analysis.

## 4. Discussion

Our group of patients is a representative sample of DTC, with baseline characteristics which are comparable to previous publications [20–24]. Typical long-term survival [25, 26] is also represented here, since no patient died due to the disease after 9.5 years of mean follow-up. Because of this observation, an association of body size measures with survival could not be done. Then, prognosis assessment was based on the frequency of disease-free state compared to persistent disease state. In this sense, body size measures were not related to higher frequency of persistent disease in the general analysis. This finding corroborates previous authors' conclusions [10, 12, 27, 28]. In one of those studies, a Korean group evidenced that body weight, height, and BMI were not associated with PTC aggressiveness or recurrence after observation for 23.6 months (1.9 year) [27]. In contrast, Trésallet et al. showed that obese patients had an increased risk of developing persistent disease [11]. Kim et al. also showed that higher body surface area (BSA) was associated to the occurrence of multiple tumor nodules in women, but not in men [9].

Regarding survival rates, few patients deceased in all those published studies and survival analysis could not be properly done, such as in our study [9, 10]. For example, in the study by Kim et al., the analysis of survival rate was jeopardized because only one death was reported in the median follow-up of 52.3 months (4.3 years) [9].

Although our sample size was smaller than those in some previous studies, our follow-up time was the longest (9.5 years). Even so, it was not possible to observe an effect of body size measures in prognosis. This could be explained because DTC mortality rates are generally much lower than other tumors [8]. Then we can conclude that sample sizes and follow-up should be much bigger to better evaluate that, but DTC is a relatively rare tumor. Therefore, we suggest that larger multicentric studies, involving more patients and longer follow-up, should be done to draw a final conclusion about the general relationship between body size measures and prognosis of DTC.

Despite that, we found one important significant association regarding body size measures and one DTC prognostic factor in our study, which was the link between higher weight and BSA with higher chance of extrathyroid tumor invasion in patients aged <45 years old. The study led by Kim et al. evidenced association between BMI and extrathyroid invasion, by showing that a 5 kg/m^2^ increase in BMI was associated with microscopic extrathyroid invasion (odds ratio of 1.23), independent of age and gender [12]. Other South Korean group was also able to demonstrate a significant relationship between extrathyroidal invasion and higher BMI in women, but not to higher BSA [9]. In line with this, Grani et al. also found BMI as a predictor of microscopic extrathyroidal extension in a cohort of 432 patients. However, the group of Paes et al. did not find an association of BMI with extrathyroidal invasion [10], and the studies by Trésallet et al. and Kim et al. did not analyze such specific feature [11, 27]. Extrathyroid invasion is a well-established factor of worse prognosis, and it is associated to higher risk of tumor recurrence and persistence and also to higher DTC-associated mortality rate [20, 25].

The mechanism for the relationship between higher body size and extrathyroid invasion, which is one feature associated to bad prognosis, could maybe be explained by the same factors that have been hypothesized to explain the association between obesity and thyroid cancer. One of them is the increased serum level of TSH, frequently observed in obese patients, since TSH is a growth factor for thyroid cells and a predictor of malignancy in thyroid nodules [29]. Other implicated factors could be hyperinsulinemia related to obesity, leading to higher bioavailable free insulin-like growth factor-1 (IGF1) levels, which, in turn, would favor tumor formation and progression [30]. Obesity is also linked to an inflammatory state, and it seems that the relationship between adipose tissue and thyroid tumorigenesis is related to the adipocytes' ability to produce immunomodulatory molecules, such as increase of tumor necrosis factor alpha (TNF*α*) with concomitant state of TNFα resistance and increase in interleukin-6 (IL-6) and leptin levels [31]. Those situations have been related to the facilitation of thyroid tumor progression [31, 32].

However, the association between higher body size and higher chance of extrathyroid invasion was only evidenced in patients aged <45 years old. Such occurrence could be explained by the fact that older patients often present other features associated with higher chances of extrathyroid invasion, which reduces the power of influence of body size measures on the feature in this age group. It is already known that older patients have a higher risk for extrathyroid invasion compared to younger patients [33]. Then, in younger patients, in the absence of other risk factors determining higher chances of extrathyroid invasion, body weight and BSA could exert a more prominent effect in determining such tumor characteristic.

Other finding in our analysis was the greatest weight loss over time in women presenting poorly differentiated tumors and in patients aged 45 years old or more who presented distant metastasis during follow-up. Both cases reveal the association of more aggressive cases of DTC and reduction of weight. Our study is the only one that evaluated this aspect of weight change over time, and, therefore, it is not possible to make a comparison with previous results [9–12, 27]. It is known that patients with advanced stages of different kinds of neoplasia present involuntary weight loss as a consequence of the decrease in nutrients ingestion or cancer-related cachexia [34]. Although such effects are not typically described in DTC cohorts, this could be one explanation for our findings. Different factors have already been associated with weight loss in DTC patients, such as gastrointestinal symptoms, appetite loss, and nausea due to radioiodine treatment [35, 36]. But such symptoms occur in short-term period after radioiodine treatment, and we did not assess in our study if those groups of patients with more aggressive tumors and weight loss had been submitted to radioiodine right before the last register of body weight in the files. Therefore, this hypothesis cannot be proved. Another possible reason for weight loss could be the use of high dose levothyroxine aiming to suppress thyroid-stimulating hormone (TSH) as part of the treatment of more aggressive DTC [14]. However, a study by Polotsky et al. [37] showed that there were no effects on body weight of patients under TSH suppression for DTC in 3–5 years of follow-up. This finding was confirmed by Samuels et al. [38], demonstrating that TSH suppression did not change body composition of women. Considering all the facts, the association between aggressiveness of the tumor and weight loss on follow-up were not consistent in all groups and is not easily explained by pathophysiology of DTC, which makes it difficult to come to definite conclusions. But since this is a newly reported finding in the field of DTC, it raises a sign to be better assessed in future dedicated studies.

## 5. Conclusions

The present study found weight and BSA to be associated with a higher chance of extrathyroidal tumor invasion in patients aged <45 years old. Nevertheless, it was not possible to observe the influence of body size on DTCs prognosis or evolution. Considering extrathyroidal tumor invasion is an important risk factor for DTC-related mortality, younger patients with higher weight and BSA should be carefully evaluated and treated to avoid worse evolution of the disease.

On the other hand, findings related to tumor aggressiveness and weight loss in specific groups deserve further mechanistic studies, since it is an unprecedented and unexpected report in the field of DTC.

## Figures and Tables

**Table 1:** Main patients' characteristics.

Characteristic	Result
Female sex, *n* (%)	296 (87.83%)
Smoking history, *n* (%)	98 (29.34%)
Caucasian, *n* (%)	271 (82.12%)
Family history of DTC, *n* (%)	13 (3.86%)
Anti-thyroid peroxidase antibodies detected at diagnosis, *n* (%)	16 (23.19%)
Anti-thyroglobulin antibodies detected at diagnosis, *n* (%)	15 (20.27%)
Lymph nodes metastasis at diagnosis, *n* (%)	88 (26.75%)
Distant metastasis at diagnosis, *n* (%)	15 (4.57%)
TNM stage I, *n* (%)	200 (61.92%)
TNM stage II, *n* (%)	48 (14.86%)
TNM stage III, *n* (%)	43 (13.31%)
TNM stage IV, *n* (%)	32 (9.91%)
Distant metastasis on evolution, *n* (%)	10 (2.97%)

**Table 2:** Main tumor features.

Feature	Result
PTC histology, *n* (%)	301 (89.29%)
FTC histology, *n* (%)	36 (10.71%)
Classical PTC, *n* (%)	124 (41.89%)
Tall cells variant PTC, *n* (%)	37 (12.50%)
Oxyphilic variant PTC, *n* (%)	11 (3.72%)
Follicular variant PTC, *n* (%)	106 (35.81%)
Other PTC variants, *n* (%)	18 (6.08%)
Unifocal tumor, *n* (%)	192 (57.31%)
Vascular and lymphatic invasion, *n* (%)	56 (16.82%)
Capsular invasion, *n* (%)	77 (23.12%)
Extrathyroid invasion, *n* (%)	70 (21.02%)
Well-differentiated tumor, *n* (%)	314 (92.29%)
Moderately differentiated tumor, *n* (%)	14 (4.20%)
Poorly differentiated tumor, *n* (%)	5 (1.5%)

**Table 3:** Significant findings from a comparison of categorical variables and anthropometric data.

Assessed characteristic	Variable	Mean ± SD	Median	*p* value
Extrathyroid invasion (< 45 years old)				*p* value no × yes^∗^
No (*N* = 111)	Initial weight (kg)	70.17 ± 14.52	67	0.0253
	Initial BSA (m^2^)	1.74 ± 0.18	1.74	0.0096
	Last weight (kg)	73.33 ± 14.48	72	0.0307
	Last BSA (m^2^)	1.77 ± 0.18	1.76	0.0105
Yes (*N* = 26)	Initial weight (kg)	78.18 ± 17.98	79.5	
	Initial BSA (m^2^)	1.85 ± 0.22	1.85	
	Last weight (kg)	82.20 ± 19.71	80.9	
	Last BSA (m^2^)	1.89 ± 0.23	1.89	

Differentiation (women)				*p* value well × poorly^†^
Well-differentiated (*N* = 278)	Weight change (kg)	+1.96 ± 6.07	+1.6	0.0306
Poorly differentiated (*N* = 5)	Weight change (kg)	−3.92 ± 5.4	−2	

Late distant metastasis (> 45 years old)				*p* value no × yes^∗^
No (*N* = 191)	Weight change (kg)	+0.67 ± 5.66	+1	0.0370
Yes (*N* = 7)	Weight change (kg)	−3.92 ± 5.4	−3	

^∗^comparison of median values by Mann–Whitney test, ^**†**^comparison of median values by Kruskal-Wallis test.

**Table 4:** Comparative analysis between prognosis and BMI categories by sex.

BMI category	Disease-free patients group	Persistent disease patients group
*Normal*		
Women, *n* (%)	60 (26%)	10 (34.5%)
Men, *n* (%)	4 (13.3%)	3 (33.3%)
*Overweight*		
Women, *n* (%)	90 (39%)	11 (38%)
Men, *n* (%)	18 (60%)	2 (22.2%)
*Obese*		
Women, *n* (%)	81 (35%)	8 (27.5%)
Men, *n* (%)	8 (26.7%)	4 (44.5%)

*P* value for comparison between categories: 0.571 (Chi-square test in women) and 0.1104 (Fisher exact test in men).

**Table 5:** Comparative analysis between prognosis and BSA quartiles by sex.

BSA quartile	Disease-free patients group	Persistent disease patients group
*Quartile 1*		
Women	58 (25%)	7 (24%)
Men	7 (23.3%)	3 (33.3%)
*Quartile 2*		
Women	53 (23%)	9 (31%)
Men	7 (23.3%)	3 (33.3%)
*Quartile 3*		
Women	61 (26.5%)	8 (27.5%)
Men	8 (26.7%)	2 (22.4%)
*Quartile 4*		
Women	59 (25.5%)	5 (17.5%)
Men	8 (26.7%)	1 (11%)

*P* value for comparison between categories: 0.6935 (Chi-square test in women) and 0.84 (Fisher exact test in men).

## Data Availability

The data used to support the findings of this study are available from the corresponding author upon request.
